# Characterisation of Groundwater Drought Using Distributed Modelling, Standardised Indices, and Principal Component Analysis

**DOI:** 10.1007/s11269-024-03997-4

**Published:** 2024-11-08

**Authors:** V. Christelis, M. M. Mansour, C. R. Jackson

**Affiliations:** British Geological Survey, Keyworth, Nottingham, NG12 5GG UK

**Keywords:** Groundwater drought, Groundwater modelling, Standardised groundwater level index, Standardised precipitation index, Principal component analysis

## Abstract

A modelling framework was developed to characterise groundwater drought at a catchment scale in the absence of adequate observational records. The framework was used to characterise historical groundwater drought events for a Chalk aquifer in southern England over the period 1971–2004 during which three major drought events occurred. A numerical groundwater model was used to simulate the groundwater level fluctuations driven by historical time-variable and spatially non-uniform recharge inputs. The standardised groundwater level index (SGI) was applied to the simulated groundwater levels to evaluate the spatial pattern of groundwater drought and of their severity and duration. A dimensionality reduction method, namely principal component analysis (PCA), was applied to the SGI dataset and to the standardised precipitation index (SPI) to further explore the spatio-temporal drought characteristics. The analysis showed inconsistency in the spatial distribution of the duration and severity among the three studied events. PCA indicated that the SPI was not a good predictor of groundwater drought during the extreme European heatwave of 2003 whereas the proposed modelling framework correctly identified the resilience of the groundwater system to that event and in line with observations. Furthermore, significant differences were observed between the spatial patterns obtained from SPI and SGI datasets highlighting the important role that hydrological and hydrogeological features of a catchment have in groundwater drought development.

## Introduction

Drought is a complex natural phenomenon which varies in space and time across catchments and can be broadly classified into three categories: meteorological drought due to a deficiency in precipitation; agricultural drought associated with reduced soil moisture; and hydrological drought when accumulated shortfalls are observed in variables such as river flows and groundwater levels (Nalbantis and Tsakiris 2009; Tsakiris et al. 2013; Hao and Singh 2015). Hydrological drought research focuses on the understanding of the response of surface water and groundwater systems to prolonged periods of less than average precipitation that occur during meteorological drought events (Van Loon 2015).

The response of groundwater systems to meteorological drought has received less attention than other types of drought for a number of reasons, some of which relate to: the complexity of aquifer systems and subsurface heterogeneity meaning that it can be challenging to produce generalised models for groundwater drought (Ojha et al. 2015); the slower response times of groundwater causing impacts to arise typically after a longer period of time (Hellwig et al. 2020); impacts not being directly visible as in the case of drying rivers (Gao et al. 2021); and the limited availability of long and reliable observational data (Mishra and Singh 2011; Han et al. 2019). Groundwater has an important role in buffering the impacts of meteorological drought by sustaining river flows and ecosystems (Kaule and Gilfedder 2021; Meyers et al. 2021; Hellwig et al. 2022), supporting water resource systems (Stigter et al. 2009) and sustaining economic activity (Suter et al. 2021).

There is increased evidence that groundwater resources are at greater risk due to climate change (Bloomfield et al. 2019; Parmesan et al. 2022; Hannaford et al. 2023; Parry et al. 2024), which highlights the importance of investigating groundwater drought dynamics both to advance knowledge and to manage impacts on society and economy (Tsakiris 2017). Groundwater drought develops as meteorological drought propagates through catchment systems resulting in persistent lower-than-normal groundwater levels (Van Loon 2015). It is generally recognised that the development of groundwater drought often lags the onset of the meteorological drought because the response of groundwater to climatic variability is strongly determined by antecedent groundwater levels, storage, and hydraulic properties of the aquifer system (Van Loon and Laaha 2015; Schreiner-McGraw and Ajami 2021).

However, the characterisation of groundwater drought is often challenging because of the limited availability of adequate observational records and the dynamic behaviour of atmospheric and hydrological variables (Bloomfield et al. 2019). In such cases, physics-based hydrological models can be used to simulate groundwater response to drought events to improve understanding. For this purpose, models of varying complexity have been applied before (e.g., Peters et al. 2005; Peters et al. 2006; Tallaksen et al. 2009; Li and Rodell 2015; Kopsiaftis et al. 2017; Seo et al. 2018). In particular, distributed groundwater models, which can represent spatially variable landscape and aquifer properties, and dynamic surface water-groundwater interactions, can simulate the spatio-temporal response of groundwater levels and storage to drought (e.g., Kang and Sridhar 2019; Hellwig et al. 2021; Bianchi et al. 2024).

Nevertheless, there are not many studies that develop frameworks based on numerical groundwater models for the characterisation of groundwater drought in catchments where observational records are either spatially sparse or temporally short. A notable example is that of Helwig et al. (2020), who developed a groundwater model of Germany and used it to test its ability to reproduce groundwater drought dynamics. Such methodologies can be particularly useful in drought-prone areas to inform groundwater resource planning and management scenarios and where groundwater level data might be difficult to collect due to cost and maintenance.

In this work we applied a comprehensive modelling framework where distributed recharge and groundwater models were used to study the propagation and characterisation of groundwater drought through an index-based analysis and a dimensionality reduction method which enable a compact representation of space-time data for multi-year analysis. The framework was applied to characterise three historical groundwater drought events in a UK groundwater catchment. The implementation uses the standardised groundwater level index (SGI) (Bloomfield and Marchant 2013) and principal component analysis (PCA) to describe the spatio-temporal patterns of groundwater drought. The results of this analysis were compared to those obtained from the standardised precipitation index (SPI) (McKee et al. 1993), which is typically used as a proxy to groundwater drought characterisation.

## Methodology

### Study Area and Data

**Fig. 1:**
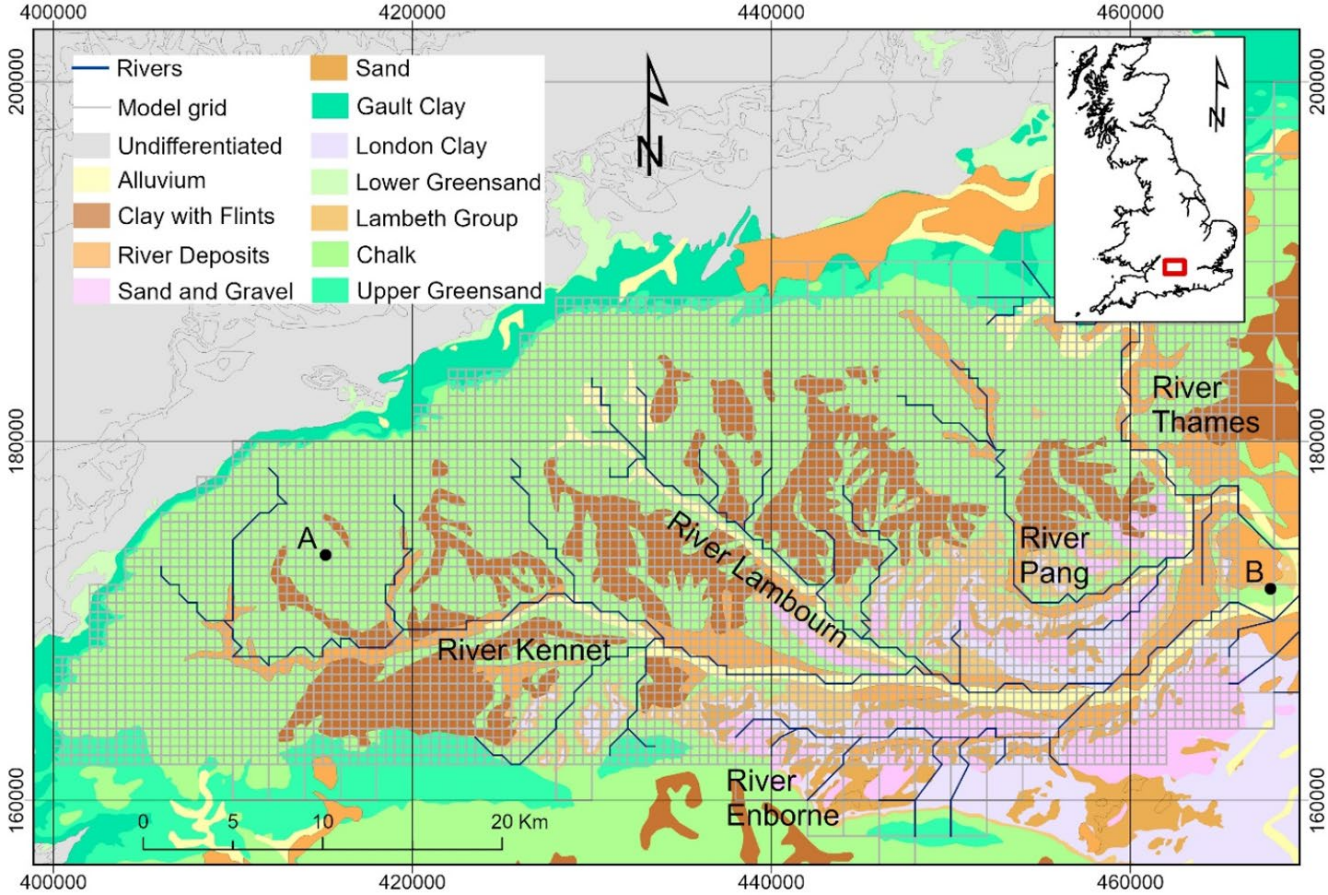
Geology of the study area overlain by groundwater model grid and river network. Contains Ordnance Survey data © Crown Copyright and database rights 2024

We studied a Chalk aquifer system in central-southern England. The Chalk aquifer, which is one of the major aquifers in the United Kingdom (Allen et al. 1997), supplies more than 70% of the water used for public supply in south-east England, and supports internationally important aquatic ecosystems (Wetherell 2023). The study area (Fig. 1) is broadly defined by the catchments of the River Kennet and River Pang, which are tributaries of the River Thames. Groundwater also discharges to neighbouring surface water catchments along the scarp slope via springs at the northern, western, and south-western edges of the Chalk outcrop. The Chalk aquifer here dips gently to the south-east below the confining London Clay (Adams 2008). Generally permeable, unconsolidated and variable clays, silts, and sands of the Lambeth Group lie on top of the Chalk and beneath the London Clay. Variably permeable Clay-with-Flints, which influence rainfall recharge, cover areas of the Chalk. Permeable alluvium and river terrace sediments are found within the valleys.

The area is predominantly rural comprising mostly arable and horticultural land and grassland. The land surface rises from approximately 37 m above sea level (m asl) at the Kennet-Thames confluence up to 270 m asl in the upper Kennet. Long-term average rainfall is approximately 725 mm year^−1^ and annual potential evapotranspiration is approximately 600 mm year^−1^. Rainfall recharge occurs across the Chalk outcrop at an average rate of approximately 225 mm year^−1^ (Mansour et al. 2018). The aquifer is unconfined, except under the London Clay in the south-east. Rivers are mostly groundwater fed with base flow indices of > 0.87. A more detailed description of the study area is provided by Jackson et al. (2011).

**Fig. 2:**
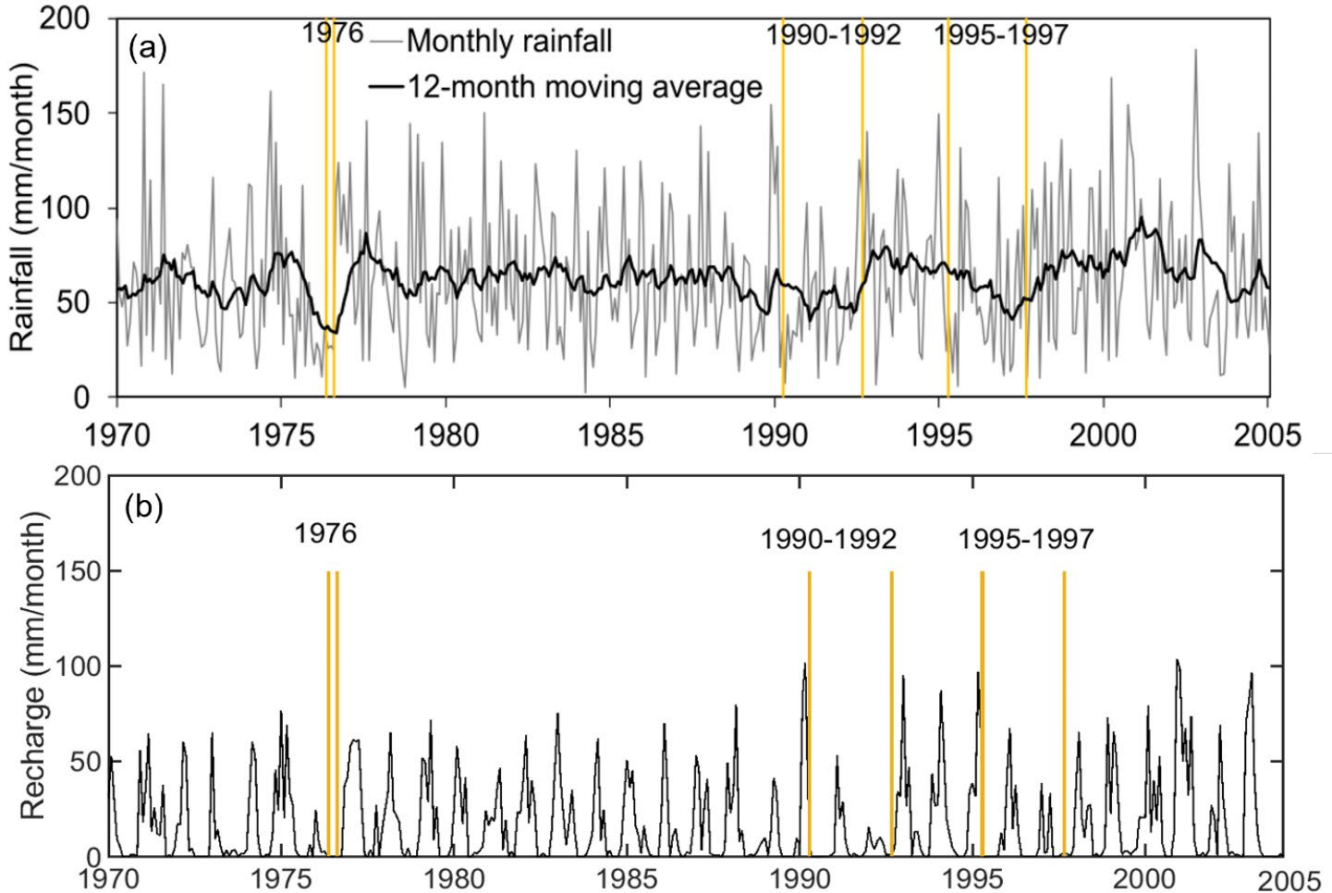
Time-series of monthly and 12-month moving average rainfall for the study area (**a**). Start and end times of the three river flow droughts as defined by Marsh et al. (2007) are also depicted. Times-series of simulated groundwater recharge over the study area is shown for the same period (**b**)

**Fig. 3:**
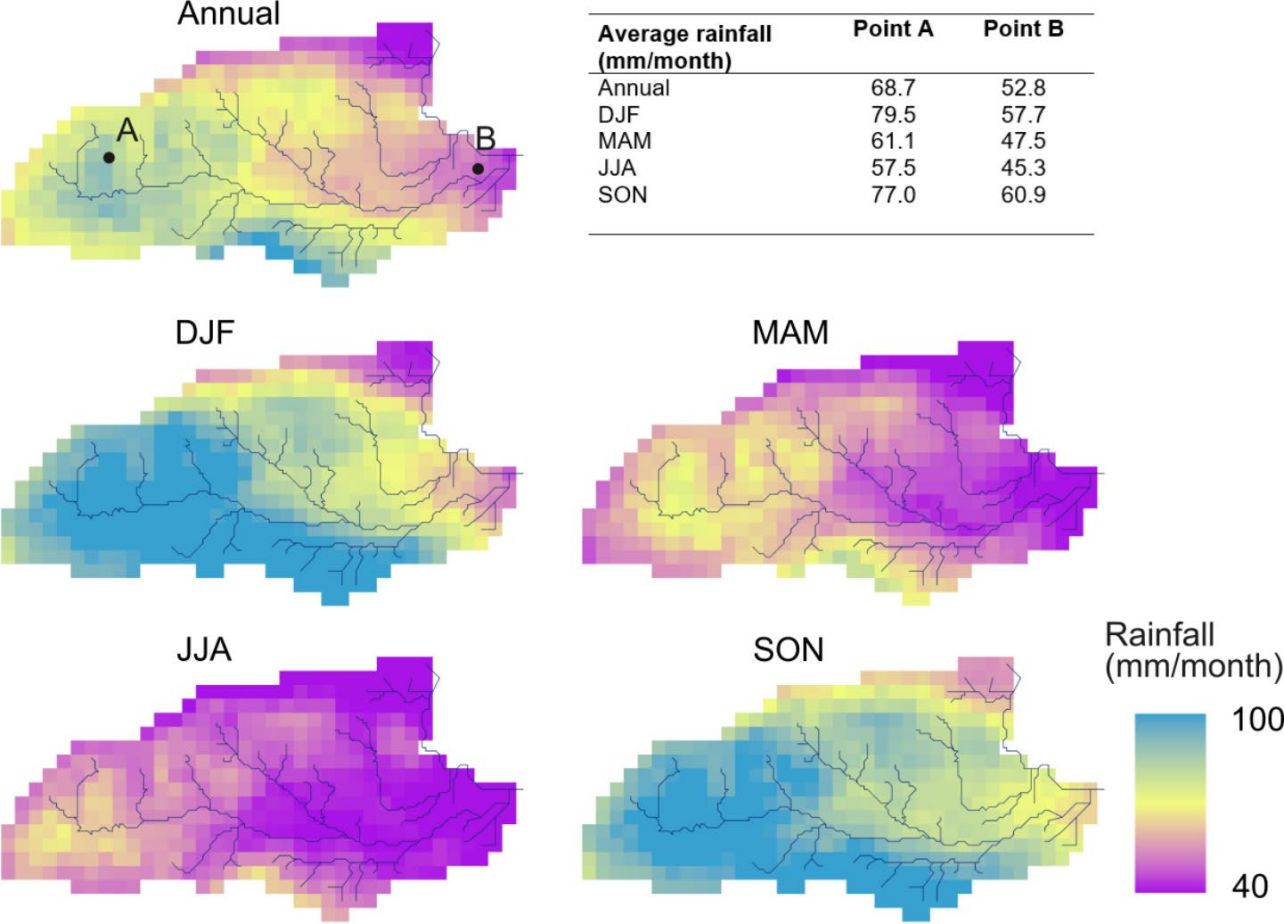
Annual average rainfall (1971–2004) and seasonal averages for winter (DJF), spring (MAM), summer (JJA), and autumn (SON) months

**Fig. 4:**
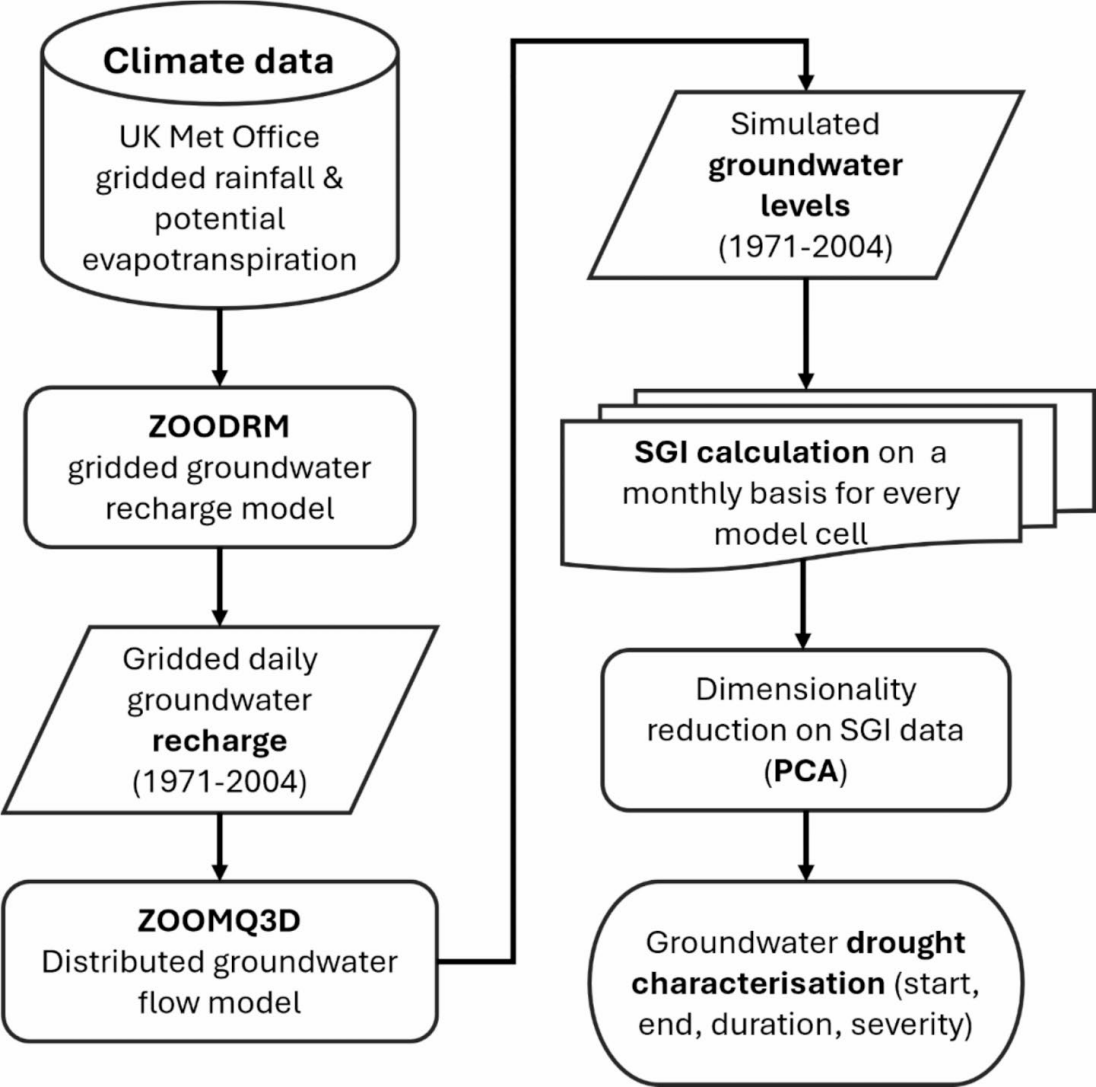
Modelling framework components

We focused on the three major drought events in the UK of 1976, 1990–1992, and 1995–1997. These are described by Marsh et al. (2007) who identified major droughts based on ranked river flow deficiencies over 9 to 24-month periods. The approximate start and end of these hydrological droughts, identified in this way, are plotted in Fig. 2a, which shows the monthly and 12-month moving average rainfall for the study area and corresponding average groundwater recharge (Fig. 2b). The 1976 drought was shorter compared to the two later multi-year drought events. In Fig. 3 rainfall is higher in the west of the area and over the interfluves between the Chalk rivers than in the east along the Thames valley. Seasonal average rainfall at the two points, A and B, shown in Fig. 1 are given in the table embedded in Fig. 4.

### Modelling Framework

Figure 4 presents a summary of the steps involved in the modelling framework while the following two subsections present the modelling tools and data that were used in more detail.

#### Distributed Modelling

Groundwater flow in the Chalk aquifer was simulated using the groundwater model instance of Jackson et al. (2011), which uses the ZOOMQ3D code (Jackson and Spink 2004). It has been previously applied to coupled land surface-groundwater modelling (Le Vine et al. 2016), to study borehole yields (Upton et al. 2019), and to study groundwater dynamics and flooding (Jackson 2012; Collins et al. 2020). It was driven by spatio-temporally varying groundwater recharge simulated with ZOODRM code (Mansour et al. 2018) over the 34-year period 1971–2004. The groundwater model uses a variable resolution Cartesian finite difference grid, which extends to the east of the River Thames; however, over the area of interest to the west of the River Thames, the grid resolution is 500 m (Fig. 1). Three layers are used to represent vertical aquifer heterogeneity. The upper layer represents the section of Chalk where hydraulic conductivity and storage are higher due to the development of fracture porosity; this is typically to a depth of 50 m below the zone of water table fluctuation (Allen et al. 1997). River-aquifer interaction is simulated using a numerical river network, and springs are included using head dependent nodes. The model was calibrated against groundwater level time-series in 207 boreholes and river flows at 20 gauging stations (Jackson et al. 2011).

#### Spatio-temporal Data Analysis

Based on simulated groundwater heads of the uppermost active model layer, a dataset of monthly SGI time-series values were calculated for each cell of the model grid. SGI applies a non-parametric normal scores transformation of the simulated levels for each calendar month. Rainfall time-series at each model grid point, calculated from the HadUK gridded 1 km resolution daily rainfall dataset (Hollis et al. 2018), were also converted into SPI values which uses a parametric method (Gamma distribution) to standardise the data. Certain threshold values can be used to identify droughts of different severity over the time-series where − 1 typically defines ‘moderate’ droughts (e.g., Tigkas et al. 2015). Also, drought events that are separated by two months or less were pooled into a single event.

To investigate the spatio-temporal dynamics of the groundwater droughts, PCA was applied to the multi-dimensional SPI and SGI datasets to derive a new set of linearly transformed uncorrelated variables which effectively describe the total variance of the original data within a much lower dimensionality (Martinez and Martinez 2010). A comprehensive description of PCA applications in earth sciences can be found in Hannachi et al. (2007). SGI values were arranged in a time-space array format with each row representing a monthly snapshot of the SGI field distributed spatially. PCA was applied to SGI dataset and the Varimax rotation technique was used to search for localised spatial patterns. PCA has been also applied on SPI values to study drought (e.g., Bonaccorso et al. 2003; Martins et al. 2012; Raziei et al. 2013; Merabti et al. 2018) and here it was implemented by using the MATLAB built-in functions (MATLAB 2023).

## Results

### Event-based Analysis

**Fig. 5:**
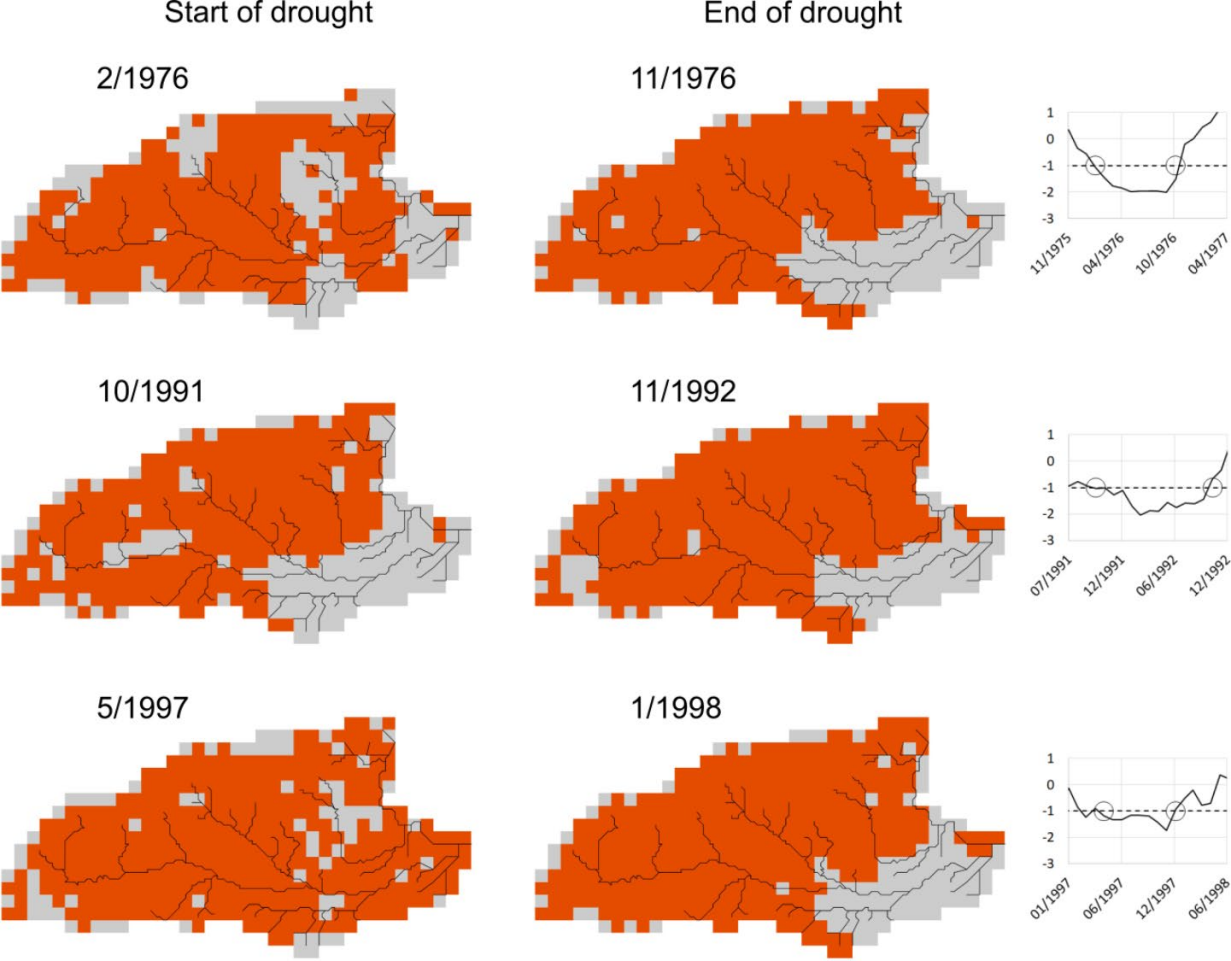
Spatial pattern of groundwater drought of 1 km grid (orange; SGI ≤ -1) and non-drought (grey; SGI > -1) areas at the start and end of the three droughts events. The right-hand plots show the time-series of the average SGI across the area, which is used to define the drought start and end times

The spatial pattern of groundwater drought across the study area at the start and end of the three events is visualised in Fig. 5. We defined the start of drought to be when the average SGI across the study area decreases below − 1, and the end to be when it recovers to values above − 1. Figure 5 shows where the SGI is below − 1 (orange pixels) and where it is above − 1 (grey pixels) at these two times. The right-hand plots in Fig. 5 show the change in the spatially averaged SGI over time during the three groundwater drought events. For brevity, we henceforth refer to these three events as the 1976, 1992, and 1997 droughts.

The most apparent feature of Fig. 5 is the different response of the confined part of the aquifer in the south-east to the larger area of unconfined aquifer. In the confined area rivers are disconnected from the Chalk aquifer by the London Clay and variations in aquifer storage are smaller. The position of the boundary between the confined and unconfined parts of the aquifer will change as groundwater levels vary in time, but the overall difference in the response is clear.

The simulations show that drought does not develop across the area in the same way at the start of each event. In 1976 the areas of the upper Lambourn and Pang catchments transition into drought later than elsewhere. In contrast most of the study area has an SGI of less than − 1 at the start of the 1997 event. At the start of the 1992 event, parts of the upper Kennet and most of the confined area have not yet transitioned into drought. The plots of the average SGI during the events show that the transition into groundwater drought was more rapid for the 1976 event, which is consistent with it being a shorter, more intense meteorological and hydrological drought than the later multi-year droughts; in November 1975 groundwater levels were above average for the time of year, the average SGI being ~ 0.4, but this had decreased to -1 by February 1976.

The spatial patterns of groundwater drought at the end of the three events, as defined by the average SGI time-series, are similar, with SGI values being less than or equal to -1 over most of the unconfined aquifer, and greater than − 1 over the confined region. The average SGI time-series show that the droughts terminated more rapidly than they developed, which likely contributes to the clear distinction between the confined and unconfined regions, and the spatial coherence across each. This is consistent with the meteorological data at the end of the droughts. The rainfall in England and Wales for September and October 1976 (313 mm) was the highest recorded for those two months (Rodda and Marsh 2011). The relatively wet summer of 1992 caused aquifer replenishment to re-commence early in the autumn and the six-month period ending in January 1993 was the wettest such sequence this century for Britain as a whole (Marsh et al. 1994).

**Fig. 6:**
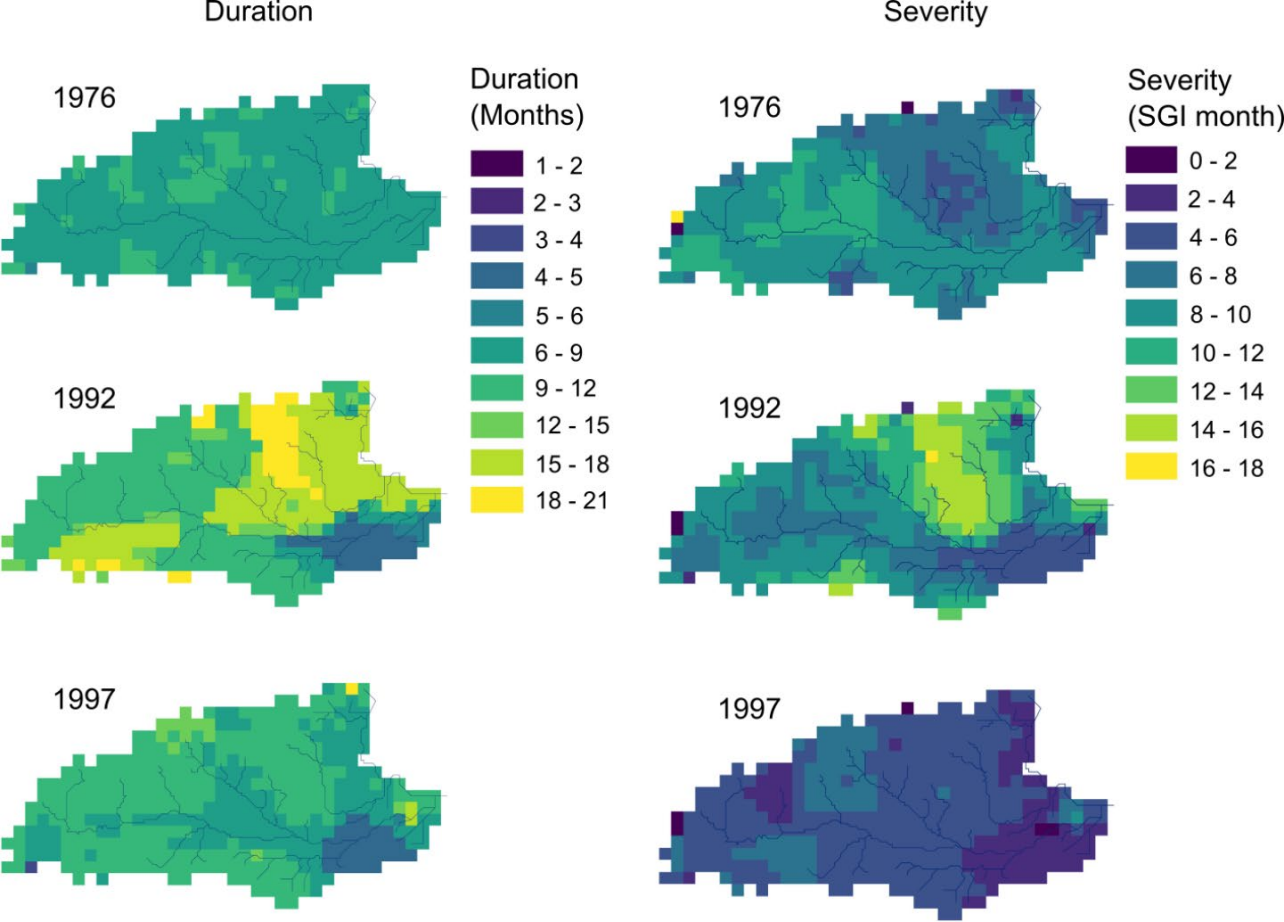
Duration and severity of the 1976, 1992, and 1997 drought events

Figure 6 shows the spatial distribution of the duration and severity of the three drought events. Severity is calculated as the sum of the SGI values (below − 1) over the duration of the drought calculated at grid points. The average duration and severity of the groundwater droughts over the study are given in Table 1. The 1992 drought was the longest of the three events at 407 days. The 1997 drought was modelled to last 296 days and the 1976 event 270 days. The simulated durations of the 1976 and 1997 droughts are relatively uniform across the area, whereas there is pronounced spatial variability in the duration of the 1992 event. The duration of the 1992 drought is significantly longer in the Pang catchment and the southern area of the upper Kennet catchment. The simulated severity of the 1992 drought (9.4 SGI months) is greater than for 1976 (8.3 SGI months) and 1997 (4.8 SGI months), due to its longer length compared to both other droughts, and higher intensity (Fig. 5 right hand plots) compared to the 1997 event. Severity values for the less intense 1997 drought are relatively uniform across the area, whereas the model simulates significant spatial variability in the severity values for the 1992 drought, with groundwater being more significantly impacted between the Lambourn and Pang catchments. In contrast, the 1976 drought was slightly more severe in the upper Kennet.

**Table 1:** Average durations and severity values calculated over the whole catchment nodes for the three drought events

Drought	Duration (days)	Severity (SGI months)
1976	270	8.3
1992	407	9.4
1997	296	4.8

### Principal Component Analysis

SPI method has been used before to approximate groundwater drought in the absence of adequate groundwater level data. Kumar et al. (2016) noted that this approach has limitations and relatively low reliability on groundwater drought prediction at regional scales. Thus, we also evaluated if this is the case for groundwater drought characterisation in our study area.

**Fig. 7:**
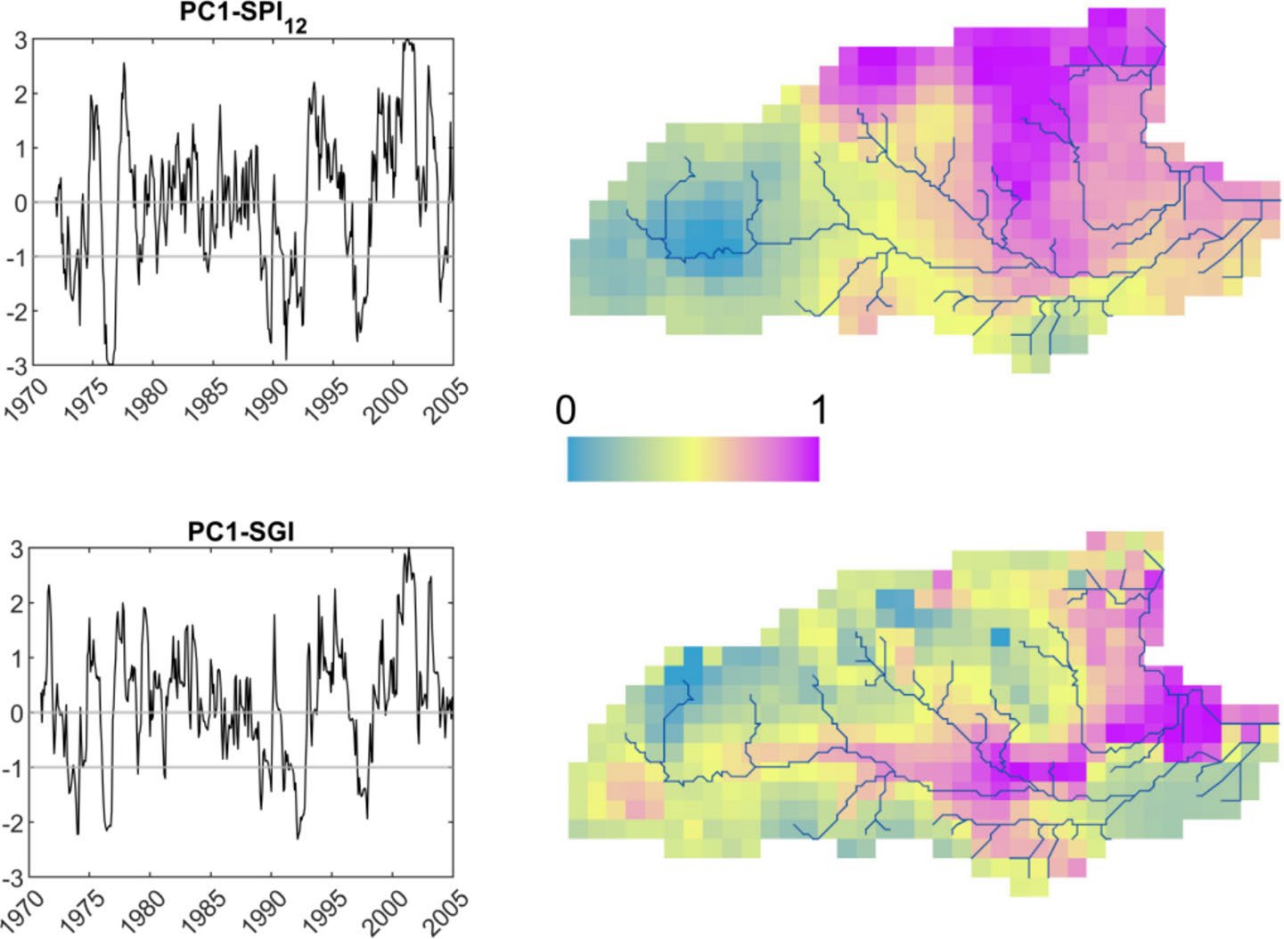
Plots of the leading principal component of SPI_12_ and SGI (left), and the corresponding rotated loadings (right)

PCA was applied to different SPI datasets (SPI_n_) by accumulating rainfall over 3, 6, 9 and 12 months. The number of principal components (PCs) retained was based on the criterion of cumulative variance explained. PC1 for all SPI datasets explained more than 90% of the total variability while the SGI space-time field resulted in the PC1 and PC2 explaining approximately 85% and 8% of the total variance, respectively. For the SGI dataset, it was recognised that PC2 mainly identified the difference response between the unconfined and the confined parts of the groundwater catchment which is a trivial finding as the confined system is much less affected by recharge variations and therefore drought events. With a focus on the PC1, a maximum cross-correlation of 0.80 was achieved between SGI-PC1 and SPI_12_-PC1 while a Spearman’s Correlation analysis returned a corresponding value of 0.689. PCA provided additional information about the spatial variation of drought in the catchment through the Varimax method that was used to generate the rotated loadings to further explore localised patterns (Fig. 7).

The temporal variability of SGI-PC1 and SPI_12_-PC1 is similar. The major meteorological droughts of 1976, 1992, and 1997 are identified by values less than the threshold of -1 in the PC1-SPI_12_ series as in the dry period of early 1973 while the rainfall deficits of 2003 are associated with the European heatwave. This variability in time is reasonably associated with the spatially averaged rainfall deficits shown in Fig. 2 but also implies that if SPI were to be used as a proxy to assess groundwater drought, then 2003 should be characterised as a significant event. Despite that the summer of 2003 was the hottest on record in Europe had a short and modest impact on UK water resources (Marsh et al. 2007) as reliably shown in PC1-SGI. Nevertheless, both leading PC scores identified the drought conditions and associated deficits across the catchment in 1973. The subsequent extreme event of 1976 is identified by PC1-SPI_12_ (values < -2) and develops into a severely dry event in the groundwater system as indicated by PC1-SGI (values of -1.5 to -2).

Despite the similarities between the PC1-SPI_12_ and PC1-SGI time-series, notable differences are apparent when these are used to characterise the spatial variability across the area. The right-hand plots in Fig. 7 show which parts of the area are most correlated with the corresponding PC1 plot on the left. For SPI_12_, high correlations are concentrated to the north-east of the river Lambourn. This area correlates strongly with the drought events, recovery phases, and wet conditions in the PC1-SPI_12_ time-series. The correlation is low in the upper Kennet catchment. In contrast to the SPI, the rotated loadings corresponding to PC1-SGI (Fig. 7 bottom right) show a spatial structure influenced by the hydrogeological system, high correlation values being associated with the Kennet, Lambourn and Thames valleys, and low values with the interfluves. The less affected confined part of the system is also identified while this spatial variability pattern provides a more focused description on the groundwater system response to droughts.

## Discussion

The simulations showed that the spatial pattern of groundwater drought differs between the three events, which is controlled by an interplay between the spatial and temporal variations in rainfall and the structure and properties of the hydrogeological system. The 1992 groundwater drought was the most severe due to its long duration and relatively high intensity, though it was not as intense as the shorter 1976 groundwater drought. Also, the 1992 groundwater drought was simulated to be less spatially uniform, being less severe in the west than the east, compared to the 1976 and 1997 droughts which were more spatially coherent.

The heterogeneity in the response of the groundwater system to drought was identified by the pattern of rotated loadings of the simulated SGI dataset. This again showed the influence of the river network and the difference between the confined and unconfined aquifer regions. This was not the case for the rotated loadings of the SPI_12_ dataset, which showed a distinct south-west to north-east difference, reflecting the influence of the land surface elevation on rainfall.

The meteorological drought associated with the European heatwave of summer 2003 was clearly identified by the SPI_12_ time-series. However, as shown by the SGI time-series, the high-temperatures during 2003 did not translate into a groundwater drought, which is known to be the case from the observations of the groundwater system. These results show that the SPI might overestimate the occurrence of groundwater droughts while being less representative of the spatial expression of groundwater drought which is in line with the findings of Kumar et al. (2016).

## Conclusions

Few studies have investigated the spatial propagation of groundwater drought in catchments where there is a lack of an adequate set of observational records. In this work, a modelling framework was developed where distributed recharge and groundwater models were used to simulate multi-year groundwater level fluctuations while the characterisation of groundwater drought and its spatio-temporal variability was based on the standardised groundwater index SGI and principal component analysis (PCA). The modelling framework was applied to a Chalk aquifer system in southern England over the historical period 1971–2004 where three major meteorological drought events have occurred. For comparison purposes, PCA was not only applied to the multi-dimensional SGI dataset but also to an SPI dataset, calculated from gridded daily rainfall observations of the area, to analyse spatial and temporal patterns of drought and evaluate possible differences.

The modelling approach was able to identify the response of the groundwater catchment to drought in line with the available information for the groundwater resources in the area over this historical period. The results also implied that when there is a paucity of groundwater level records, the use of SPI as a proxy to characterise groundwater drought might lead to overestimation of the impacts of rainfall deficits on the groundwater system. On the contrary, the developed modelling framework correctly indicated the resilience of the groundwater system to a significant meteorological drought event.

In addition, by considering the role of the hydrological and hydrogeological features of the catchment through the numerical groundwater model and in combination with PCA, specific spatial variability patterns where revealed. In overall, the study showed that for catchments with spatially sparse groundwater level data, the use of distributed groundwater models along with standardised indices and dimensionality reduction methods, such as SGI and PCA, can be a useful approach in understanding the catchment response to drought. This modelling approach can be an informative tool for groundwater managers on past and future behaviour of the system under drought conditions. Further work is needed to understand how different spatio-temporal patterns of meteorological drought interact with groundwater systems and how these patterns are controlled by the hydrological features and hydrogeological properties of aquifers at catchment scales
